# *Cistanche deserticola* Polysaccharides Protect Against Doxorubicin-Induced Cardiotoxicity via Antioxidant and Mitochondrial Mechanisms

**DOI:** 10.3390/antiox14121461

**Published:** 2025-12-05

**Authors:** Jingyi Qi, Yang Zhang, Mingyang Cui, Yufang Shi, Xinyu Luo, Chang Fan, Sitong Wan, Peng An, Yongting Luo, Junjie Luo

**Affiliations:** Department of Nutrition and Health, China Agricultural University, Beijing 100193, China

**Keywords:** DOX-induced cardiotoxicity, *Cistanche deserticola* polysaccharides, antioxidant activity, mitochondrial structure, mitochondrial respiration

## Abstract

Doxorubicin (DOX), a clinical broad-spectrum anthracycline chemotherapeutic agent, induces dose-dependent cardiotoxicity that progresses to heart failure (HF), thereby severely limiting its clinical application. Mitochondrial dysfunction and oxidative stress dysregulation are core pathological mechanisms underlying DOX-induced myocardial injury. This study aimed to investigate the protective effect and underlying mechanism of *Cistanche deserticola* polysaccharides (CDPs) against DOX-induced cardiotoxicity in C57BL/6J mice. Compared with the DOX model group, CDPs significantly increased left ventricular ejection fraction (LVEF) and left ventricular fractional shortening (LVFS), and reduced the activities of serum creatine kinase (CK), creatine kinase-MB (CK-MB), and lactate dehydrogenase (LDH). Additionally, CDPs notably decreased the malondialdehyde (MDA) levels in serum and myocardial tissue, while significantly enhancing the activities of superoxide dismutase (SOD) and glutathione peroxidase (GSH-Px). Moreover, CDPs ameliorated mitochondrial swelling and crista fracture, upregulated the expression of mitochondrial respiratory chain complex-related genes, and increased adenosine triphosphate (ATP) production. In conclusion, CDPs alleviate DOX-induced cardiotoxicity and protect cardiac function by inhibiting myocardial oxidative stress and improving mitochondrial function, which provides a potential therapeutic strategy for preventing DOX-related cardiotoxicity.

## 1. Introduction

Doxorubicin (DOX), a frontline broad-spectrum anthracycline chemotherapeutic agent, remains indispensable in the clinical treatment of diverse malignancies, including breast cancer, leukemia, and lymphoma, owing to its potent antiproliferative and cytotoxic effects [1,2,3]. However, its clinical utility is severely limited by dose-dependent and irreversible cardiotoxicity [4,5]. Accumulating evidence has clearly identified oxidative stress and mitochondrial dysfunction as core pathological drivers of DOX-induced myocardial injury [6,7]. DOX exhibits selective accumulation in myocardial mitochondria, a phenomenon attributed to the heart’s high metabolic demand and weak antioxidant capacity. Within mitochondria, DOX disrupts the mitochondrial electron transport chain (ETC) [8,9,10,11], causing massive electron leakage, triggering excessive reactive oxygen species (ROS) production that overwhelms endogenous antioxidant systems [12]. The sustained ROS overload further impairs oxidative phosphorylation (OXPHOS), induces mitochondrial structural damage, and reduces adenosine triphosphate (ATP) synthesis—depriving cardiomyocytes of essential energy [13,14]. Ultimately, this cascade drives cardiomyocyte apoptosis, promotes myocardial remodeling, and progresses to irreversible heart failure [15]. Thus, targeting mitochondrial function and oxidative stress imbalance emerges as a promising strategy to alleviate DOX-induced cardiotoxicity. In this context, natural products with both antioxidant and mitochondrial protective properties have attracted increasing attention [16,17], as they often exhibit low toxicity and multi-target effects—advantages over synthetic agents [18,19].

*Cistanche deserticola* Y.C. Ma (CD) is both a traditional tonic and an approved food ingredient in China. Its bioactive compounds have demonstrated protective effects in various models of organ damage [20,21,22,23,24]. As the key active fraction, CD polysaccharides (CDPs), with high safety and low toxicity [23], exhibit a broad spectrum of pharmacological activities, such as immunomodulation, anti-aging effects, spleen function improvement, and anti-tumor properties [24,25,26,27]. Existing studies have demonstrated that CDPs can exert neuroprotective effects by mitigating oxidative stress and regulating the DJ-1 signaling pathway [21]. They also improved mitochondrial membrane potential and restored ATP synthesis in slow transit constipation mice [22]. Given that DOX-induced cardiotoxicity is driven by both oxidative stress and mitochondrial dysfunction, we hypothesize that CDPs may alleviate DOX-related cardiac injury through dual targeting of oxidative stress and mitochondrial function.

In this study, we established a DOX-induced cardiotoxicity model in C57BL/6J mice to explore the cardioprotective effect of CDPs. We evaluated cardiac function, myocardial oxidative stress markers, and mitochondrial structural/functional parameters, aiming to clarify the potential mechanism of CDPs in preventing DOX-related cardiotoxicity and providing a novel candidate for clinical application.

## 2. Materials and Methods

### 2.1. Chemicals and Reagents

DOX (HPLC ≥ 99%) was purchased from Solarbio Life Science Co., Ltd. (Beijing, China; Cat: D8740). CDPs (purity ≥ 98% by the phenol–sulfuric acid method) were obtained from Beijing Kangruina Biotechnology Co., Ltd. (Beijing, China, Cat: LA3617).

### 2.2. Identification of CDPs

The monosaccharide composition of CDPs was analyzed via high-performance liquid chromatography (HPLC; Shimadzu LC-20AD, Kyoto, Japan). Briefly, 2 mg of CDPs was mixed with 3 mL of 2 M trifluoroacetic acid (TFA) in a sealed tube, followed by hydrolysis at 120 °C for 3 h. After cooling, methanol was added to the sample, and evaporation was repeated three times to eliminate residual TFA.

For derivatization, 250 μL of 0.6 mol/L NaOH and 500 μL of 0.4 mol/L 1-phenyl-3-methyl-5-pyrazolone (PMP) in methanol were added to either the hydrolysate or 1 mg/mL standard monosaccharide mixture. The mixture was reacted at 70 °C for 1 h and cooled in cold water for 10 min, and then 500 μL of 0.3 mol/L HCl was added to stop the reaction. After three extractions with chloroform, the supernatant was subjected to HPLC analysis. HPLC separation was performed on an Xtimate-C18 column (4.6 mm × 200 mm, 5 μm) at 30 °C, using a mobile phase of acetonitrile and 0.1 M phosphate buffer (pH 6.7) at a 17:83 (*v*/*v*) ratio, with a flow rate of 1 mL/min. The same chromatographic conditions were applied to monosaccharide standards. The monosaccharide content in CDPs was calculated by comparing the peak areas of the sample with those of the standards, and the molar ratio of monosaccharides was determined via the external standard method.

The molecular weight of CDPs was measured using high-performance gel permeation chromatography (HPGPC). For functional group analysis, 1 mg of CDPs was ground with 100 mg of dried KBr powder, pressed into thin pellets, and scanned with a Nicolet 6700 Fourier transform infrared spectroscopy (FTIR) spectrometer (Thermo Fisher Scientific, Waltham, MA, USA) over the wavenumber range of 4000–400 cm^−1^.

### 2.3. Animals and Experimental Design

A total of 42 male C57BL/6 mice (6 weeks old, 20–25g) were purchased from SLAC Laboratory Animal Co., Ltd. (Changsha, China). Mice were housed under specific pathogen-free conditions (temperature: 20–26 °C; humidity: 40–70%; pressure: 45 Pa; animal illumination: 15–20 Lux; light: 12 h/12 h light/dark cycle). After 1 week of acclimatization, mice were randomly divided into three groups. Control Group (Ctrl) (*n* = 14): Saline was administered for a duration of 3 weeks, following the administration protocol established for the prevention group. Model Group (DOX) (*n* = 14): Mice received intraperitoneal injections of DOX (dissolved in normal saline) at a dose of 3 mg/kg every other day, for a total of 7 administrations [14,28,29]. Prevention Group (CDPs) (*n* = 14): CDPs were administered intragastrically at a dosage of 200 mg/kg/day for 3 weeks. Concurrently, DOX was administered following the modeling protocol after the 1 week of intragastric administration. The CDPs dose was determined based on a previous study (Li, J., et al., 2025), and the dose has been studied and found to have no significant effect on the growth of mice and not to induce an oxidative stress response [20].Three days after the final DOX injection, mice underwent echocardiographic evaluation and then were sacrificed for subsequent experiments. Euthanasia was performed via inhalation of 2% isoflurane followed by cervical dislocation. Body weight, heart weight, and tibial length were measured immediately after euthanasia. Heart tissues and blood samples were collected for histopathological examination, biochemical assays, and mechanism-related analyses.

### 2.4. Echocardiography and Hemodynamics

Cardiac geometry and function were assessed using a Vevo 3100 High-Resolution In Vivo Micro-Imaging System (FUJIFILM VisualSonics, Toronto, ON, Canada). Mice were anesthetized with 1.5% isoflurane and placed in a supine position on a heating table. Heart rate was maintained at 450–500 beats per minute (bpm) whenever possible. Left ventricular (LV) echocardiography was performed in parasternal long-axis and short-axis views at a frame rate of 233 Hz. LV ejection fraction (LVEF), LV fractional shortening (LVFS), left ventricular internal dimension at systole (LVID; s), LV end-systolic volume (LVESV), and cardiac output (CO) were calculated based on LV dimensions at end-systole and end-diastole. All experiments and data analyses were performed by investigators blinded to the experimental groups.

### 2.5. Measurement of Serum Myocardial Enzymes

The whole blood sample is placed at room temperature in a 2 mL tube with a clot activator (X0021, Xinkang Medical Equipment Co., Ltd, Taizhou, China) for 2 h, followed by centrifugation at 3000 rpm for 15 min under a temperature-controlled condition of 2–8 °C. The supernatant obtained from centrifugation was collected and designated as serum. Serum levels of creatine kinase (CK) and lactate dehydrogenase (LDH) were determined using commercial assay kits (S03024 and S03034; Rayto, Shenzhen, China). Serum creatine kinase-MB (CK-MB) was determined using a commercial assay kit (C060, Changchun Huili, Changchun, China).

### 2.6. Histological Examination

After echocardiography, hearts were perfused with ice-cold 0.1 M PBS (pH 7.4), fixed overnight in 4% paraformaldehyde (pH 7.4), embedded in paraffin, and serially sectioned into 5 μm thick slices. Sections were stained with hematoxylin and eosin (H&E; G1120, Solarbio, Beijing, China) for routine histological observation under a light microscope. Sirius Red staining (G1472, Solarbio, Beijing, China) was used to evaluate collagen deposition. Wheat germ agglutinin (WGA) staining (W11261, Thermo Fisher Scientific, Waltham, MA, USA) was performed to measure the cardiomyocyte cross-sectional area. Histological images were acquired using a Zeiss Axioplan2 light microscope (Zeiss, Oberkochen, Germany). For quantitative analysis, three adjacent sections from each mouse were analyzed using ImageJ software (version 1.52, National Institutes of Health, Bethesda, MD, USA).

### 2.7. Biochemical Assays

The levels of malondialdehyde (MDA) and the activities of superoxide dismutase (SOD) and glutathione peroxidase (GSH-Px) in serum and heart tissues were measured using commercial assay kits (MDA: Cat. No. A003-1-2; SOD: Cat. No. A001-3-2; GSH-Px: Cat. No. A005-1-2; Nanjing Jiancheng Technology, Nanjing, China) according to the manufacturers’ instructions. A microplate reader (Thermo Fisher Scientific, Waltham, MA, USA) was used to detect absorbance at 532 nm (MDA), 450 nm (SOD), and 412 nm (GSH-Px), respectively.

### 2.8. Transmission Electron Microscopy (TEM)

Freshly collected heart tissues were immediately fixed by immersion in 2% glutaraldehyde and 2.5% paraformaldehyde in 0.1 M sodium cacodylate buffer (pH 7.4) for at least 1 h at room temperature, followed by overnight fixation at 4 °C. Fixed samples were dehydrated using a gradient ethanol series (30%, 50%, 70%, 90%, 100%). After dehydration, fixed samples were infiltrated and embedded in epoxy resin (Epon 812). Resin-sample mixtures were degassed in a vacuum desiccator to eliminate air bubbles and then polymerized at 60 °C for 24–48 h to form solid blocks. Ultrathin sections (80–90 nm thickness) were sliced using the Leica Ultracut E ultramicrotome (Leica Microsystems, Wetzlar, Germany), stained with 2% uranyl acetate and lead citrate (Sigma-Aldrich, St. Louis, MO, USA) for contrast, and imaged using a JEOL 1400 transmission electron microscope (Japan Electron Optics Laboratory Co., Ltd., Peabody, MA, USA) equipped with a Gatan Orius SC1000 digital CCD camera (Gatan, Pleasanton, CA, USA). The acquired images were quantitatively analyzed using ImageJ software (v1.54f, NIH, Bethesda, MD, USA).

### 2.9. RNA Extraction and Real-Time Quantitative PCR (RT-qPCR)

Total RNA was extracted from heart tissues using TRIzol reagent (Thermo Fisher Scientific, Cat. No. 15596026) according to the manufacturer’s protocol. RNA purity was assessed using a Nanodrop spectrophotometer (Thermo Scientific) at 260 nm/280 nm. Subsequently, 1 μg of total RNA was reverse-transcribed into cDNA using Reverse Transcription Reagent kits (FT301, Vazyme, Nanjing, China). RT-qPCR was performed using SYBR Green Master Mix (Cat. No. Q711-02, Vazyme,) on an Applied Biosystems StepOnePlus Real-Time PCR System (ABI 7500, Thermo Fisher Scientific). The RT-qPCR program was set at 95 °C for 5 min, followed by 40 cycles of 95 °C for 10 s and 60 °C for 30 s according to the manufacturer’s instructions. All reactions were performed in triplicate. Before the formal experiment, we determined the specificity of the primers through melting curve analysis (Supplemental Figure S1) and analyzed the amplification efficiency (E) of RT-qPCR under this condition (Supplemental Figure S2) [30]. Results were analyzed using the comparative cycle threshold (ΔΔCt) method. Samples were normalized to *β*-actin (*Actb*) to account for cDNA loading differences. Primer sequences are provided in Supplemental Table S1.

### 2.10. ATP Quantification

Cardiac tissues were determined by the Enhanced ATP Assay Kit (S0027, Beyotime, Beijing, China) following the manufacturer’s protocol.

### 2.11. Statistical Analysis

All data are expressed as the mean ± standard deviation (SD). Significant differences among groups were analyzed using one-way analysis of variance (ANOVA) followed by Tukey’s test. All statistical analyses were performed using GraphPad Prism software (version 9.0, GraphPad Software, San Diego, CA, USA). A *p*-value < 0.05 was considered statistically significant.

## 3. Results

### 3.1. Structural Analysis of CDPs

The monosaccharide composition analysis of CDPs showed that the main components of CDPs were glucose (Glc), mannose (Man), and galactose (Gal), with a molar ratio of 2.8: 0.5: 0.18 (Supplemental Figure S3A,B and Table S2). The HPGPC analysis revealed that the CDPs exhibited a broad molecular weight distribution, and the low-/middle–low-molecular-weight components constitute the absolute majority. The high-molecular-weight fraction displayed a narrow distribution, with a number-average molecular weight (Mn) of approximately 10,316 Da and a weight-average molecular weight (Mw) of 13,314 Da, yielding a polydispersity index (Mw/Mn) of 1.29, indicative of good uniformity. The medium–low-molecular-weight fraction (Mn = 665 Da, Mw = 902 Da) constituted approximately 40.6486% of the total polysaccharide content. The low-molecular-weight fraction (Mn = 53 Da, Mw = 156 Da) constituted approximately 57.74% of the total polysaccharide content (Supplemental Figure S3C,D and Table S3).

The FTIR spectra detected from 400 to 4000 cm^−1^ of CDPs displayed characteristic bands at 596, 812, 1253, 1025, 1157, 1265, 1384, 1631, 2934, and 3425 cm^−1^ (Supplemental Figure S1E). The absorption bands at 3425 cm^−1^ and 2934 cm^−1^ were attributed to the stretching vibrations of O-H bonds and C-H bonds, which are characteristic absorption peaks of polysaccharides. The strong absorption band at 1025 cm^−1^ corresponded to the C–O–C glycosidic band vibration, indicating the presence of pyranose in the CDPs. The absorption peaks appearing at 1631 cm^−1^ were caused by the C=O stretching vibration. The major functional groups are consistent with the present study [22].

### 3.2. CDPs Attenuate DOX-Induced Cardiac Dysfunction and Cardiomyocyte Injury

**Figure 1:**
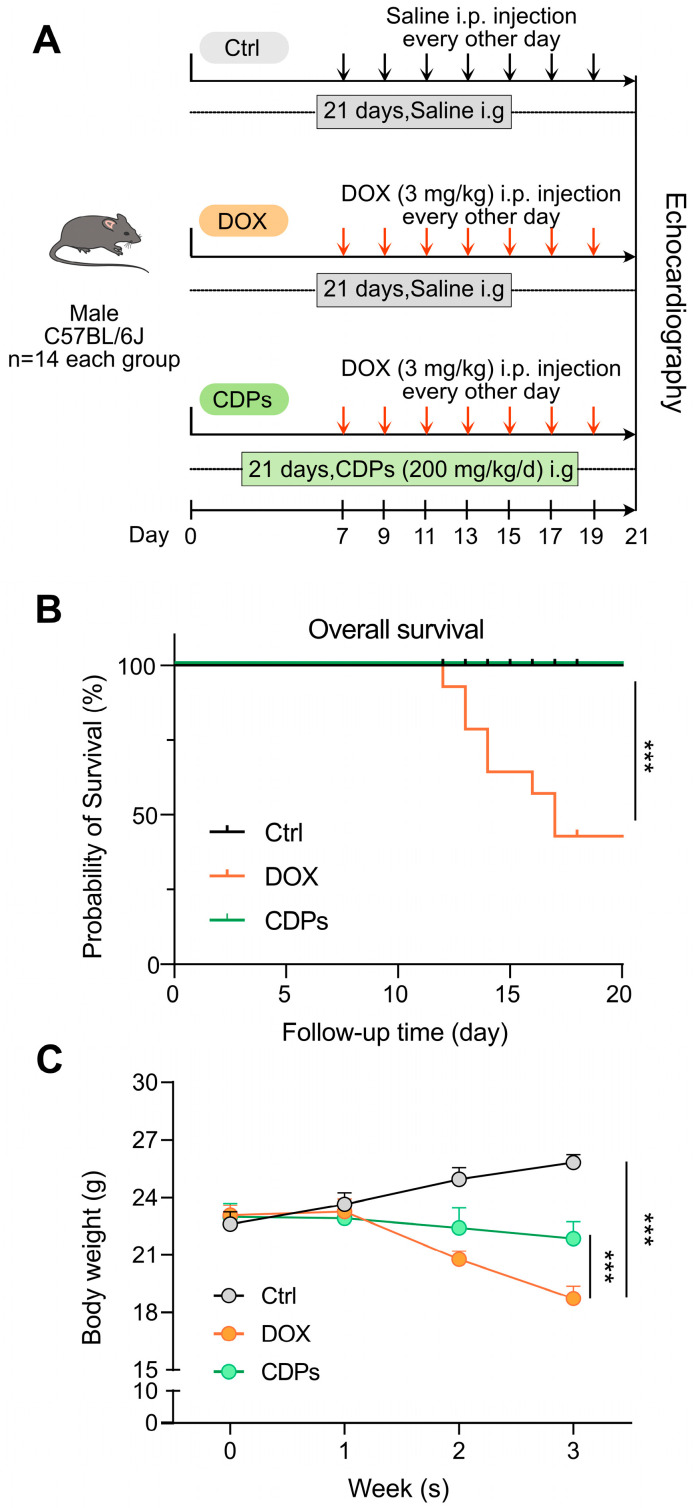
Animal experimental protocol, survival curve, and body weight changes in mice from the three groups. (**A**) Schematic diagram of the animal experimental procedure; (**B**) survival curves of the mice in the three groups (*n* = 14); (**C**) body weight changes of mice in the three groups (*n* = 6). All data are presented as mean ± standard deviation (SD). Statistical analysis was performed with one-way ANOVA. *** *p* < 0.001.

To investigate the protective effect and underlying mechanism of CDPs against DOX-induced heart failure in C57BL/6J mice, the mice were randomly assigned to three groups: the control group, the DOX model group, and the CDPs-treated group (Figure 1A). DOX mice showed significantly increased mortality and decreased body weight compared to control mice. However, CDPs treatment reduced DOX-induced mortality and body weight loss (Figure 1B,C).

**Figure 2:**
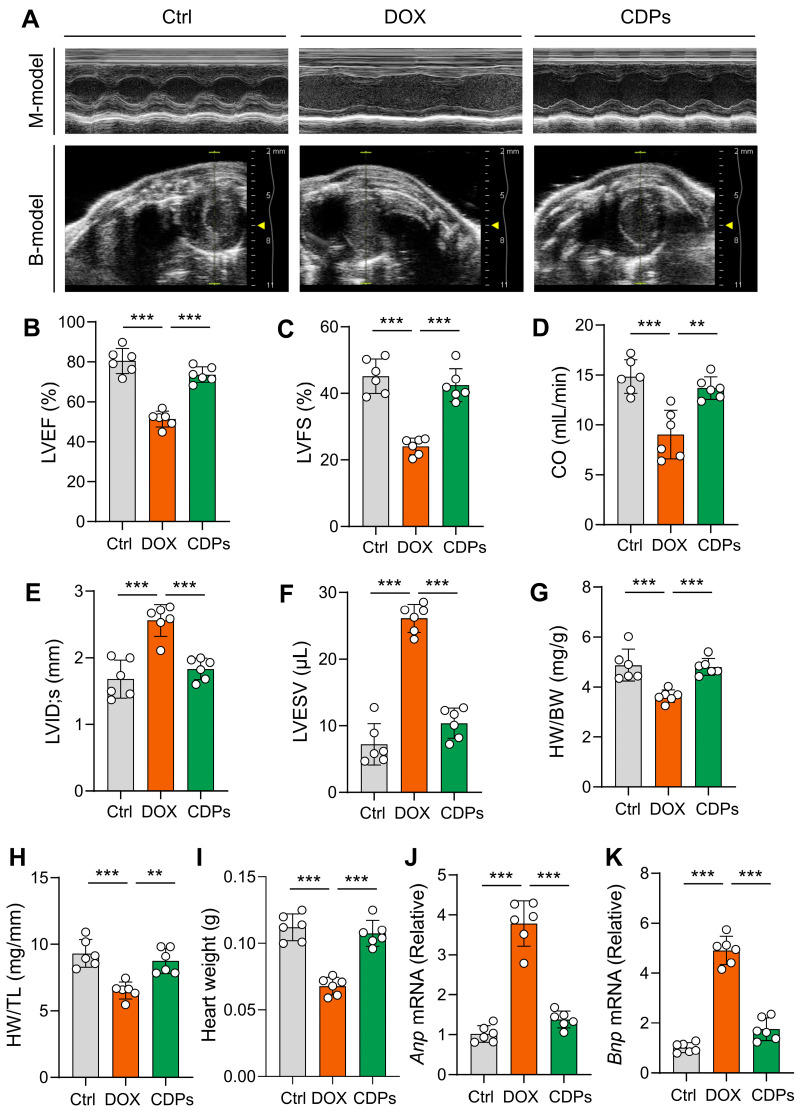
CDPs attenuate DOX-induced cardiac dysfunction and cardiomyocyte injury. (**A**) Representative B- and M-mode echocardiographic images of the heart; (**B**–**F**) echocardiographic parameters: left ventricular ejection fraction (LVEF, **B**), left ventricular fractional shortening (LVFS, **C**), cardiac output (CO, **D**), left ventricular internal dimension at systole (LVID; s, **E**), and left ventricular internal dimension at end-diastole (LVESV, **F**) (*n* = 6); (**G**) heart weight/body weight (HW/BW) ratio (*n* = 6); (**H**) heart weight/tibia length (HW/TL) ratio (*n* = 6); (**I**) heart weight (*n* = 6); (**J**,**K**) relative mRNA expression levels of atrial natriuretic peptide (*Anp*, **J**) and brain natriuretic peptide (*Bnp*, **K**) in the heart (*n* = 6). All data are presented as mean ± SD. Statistical analysis was performed with one-way ANOVA. ** *p* < 0.01, *** *p* < 0.001.

Cardiac echocardiography showed that CDPs treatment significantly improved DOX-induced cardiac dysfunction in mice, as evidenced by increased LVEF, LVFS, and CO, as well as decreased LVID; s and LVSEV (Figure 2A–F).

Compared with the DOX-treated group, the heart weight, heart weight-to-body weight ratio (HW/BW), and heart weight-to-tibial length ratio (HW/TL) were significantly increased in the CDPs-treated group (Figure 2G–I), indicating that CDPs could mitigate DOX-induced cardiomyocyte loss and restore myocardial mass. Meanwhile, the elevated mRNA levels of *Anp* and *Bnp* (classic molecular markers of pathological cardiac remodeling) in the DOX group were significantly reduced by CDPs treatment (Figure 2J,K), further confirming that CDPs attenuated DOX-induced pathological cardiac remodeling. These results collectively demonstrate that CDPs could ameliorate DOX-induced cardiac dysfunction in mice.

### 3.3. CDPs Alleviate the Histological Changes of Cardiac Tissue of Mice with DOX-Induced Injury

**Figure 3:**
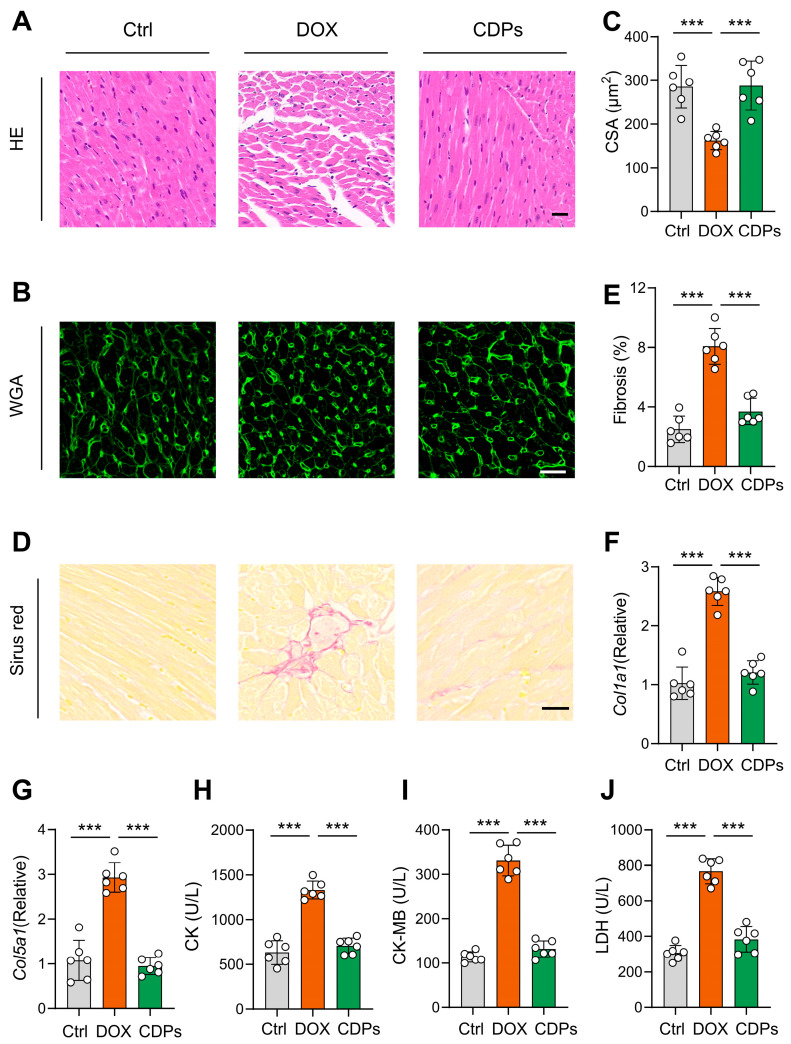
CDPs alleviate the histological changes in the cardiac tissues of DOX-induced injured mice. (A) Representative histopathological cross-sectional images of mice hearts (scale bar: 20 μm); (**B**,**C**) wheat germ agglutinin (WGA) staining for the detection of myocardial hypertrophy (**B**) and quantitative analysis (**C**) (scale bar: 20 μm; *n* = 6, at least 10 sections per mouse); (**D**,**E**) Sirius Red staining for the detection of myocardial fibrosis; (**D**) and quantification of fibrosis fraction (**E**) (scale bar: 50 μm; *n* = 6, at least 10 sections per mouse); (**F**,**G**) relative mRNA expression levels of *Col1a1* (**F**) and *Col5a1* (**G**) in the heart (*n* = 6); (**H**–**J**) serum levels of creatine kinase (CK, (**H**)), creatine kinase-MB (CK-MB, (**I**)), and lactate dehydrogenase (LDH, (**J**)) (*n* = 6). All data are presented as mean ± SD. Statistical analysis was performed with one-way ANOVA. *** *p* < 0.001.

H&E staining revealed the loss of myocardial orientation and structural disorganization in DOX-treated mice. Sirius Red staining showed a slight increase in myocardial fibrosis area in DOX-treated mice compared to the control group. Moreover, WGA staining indicated that DOX treatment significantly reduced cardiomyocyte size. However, CDPs alleviated these histological changes in the cardiac tissue of DOX-induced injured mice (Figure 3A–E). Meanwhile, the mRNA levels of *Col1a1* and *Col5a1* (key genes involved in collagen synthesis) also confirmed that CDPs significantly attenuated DOX-induced myocardial fibrosis (Figure 3F,G).

Subsequently, myocardial injury was evaluated by measuring serum levels of CK, CK-MB, and LDH. Consistent with the histological staining results, DOX treatment significantly increased the levels of these early myocardial injury biomarkers in mice, and these detrimental effects were notably mitigated by CDPs (Figure 3H–J).

### 3.4. CDPs Treatment Inhibited DOX-Induced Oxidative Injury

**Figure 4:**
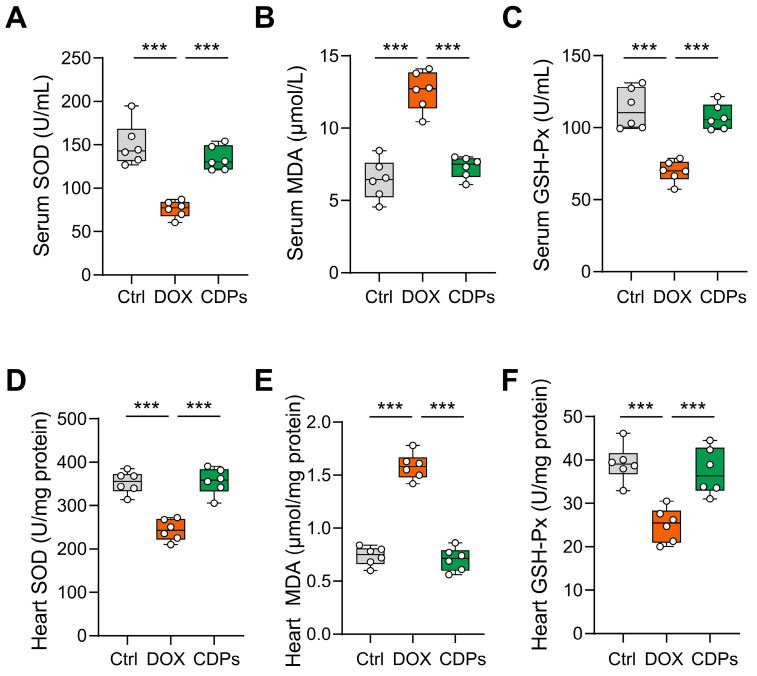
CDPs improve oxidative stress levels in mice with DOX-induced heart failure. (**A**–**C**) Serum levels of superoxide dismutase (SOD, (**A**)), malondialdehyde (MDA, (**B**)), and glutathione peroxidase (GSH-Px, (**C**)) (*n* = 6); (**D**–**F**) heart tissue levels of SOD (**D**), MDA (**E**), and GSH-Px (**F**) (*n* = 6). All data are presented as mean ± SD. Statistical analysis was performed with one-way ANOVA. *** *p* < 0.001.

Previous studies have shown that CDPs are natural antioxidants with significant anti-free radical properties [22,31]. To evaluate the efficacy of CDPs in preserving normal cardiac function and counteracting DOX-induced oxidative stress, we measured the activities of antioxidant enzymes and the levels of oxidative stress markers in the cardiac tissue and serum of DOX-treated mice. Compared with the control group, DOX-induced injured mice showed increased MDA content in myocardial tissue, along with significantly decreased activities of SOD and GSH-Px. However, CDPs upregulated SOD activity in both cardiac tissue and serum, downregulated MDA levels, and enhanced GSH-Px activity in DOX-induced cardiomyopathy mice—all with statistically significant differences compared with the DOX group (*p* < 0.001) (Figure 4A–F).

### 3.5. CDPs Alleviate DOX-Induced Cardiomyopathy Through Improving Mitochondrial Function

DOX-induced oxidative stress relies heavily on mitochondria: mitochondria produce excessive ROS after DOX exposure and are also primary targets of ROS damage [32,33,34]. Given mitochondria’s pivotal role in cardiomyocyte survival, we explored whether CDPs alleviate DOX cardiotoxicity by restoring mitochondrial structure and function.

**Figure 5:**
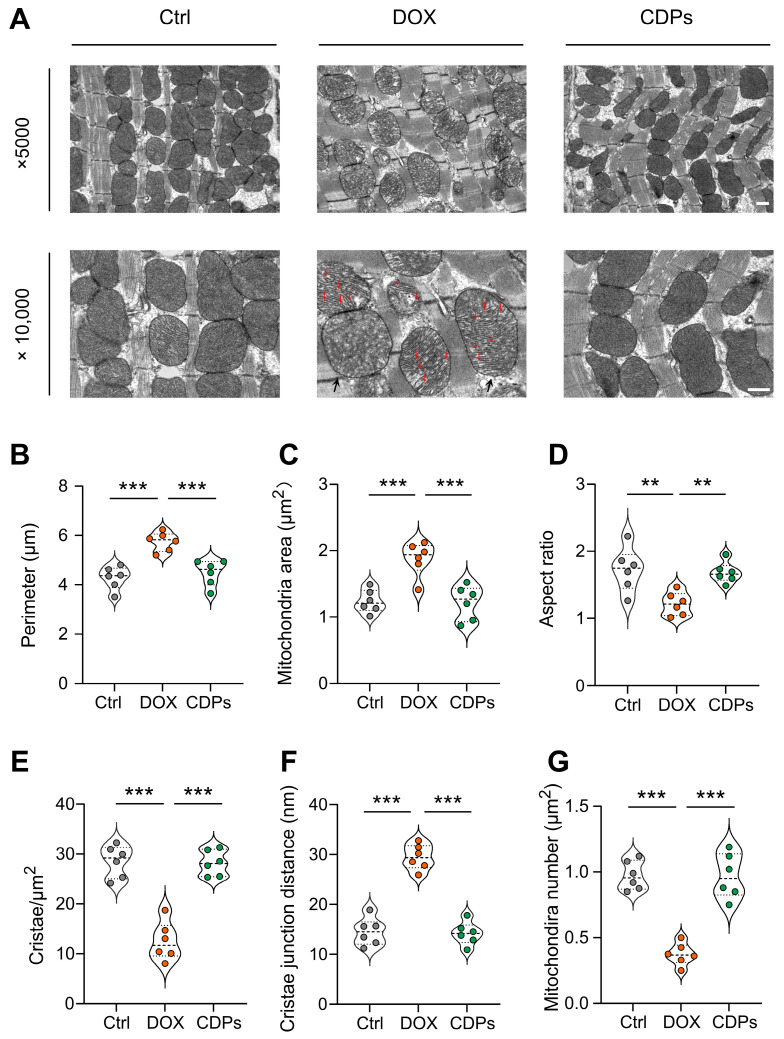
CDPs ameliorate DOX-induced myocardial mitochondrial structural damage. (**A**) Representative transmission electron microscopy (TEM) images of cardiac mitochondria (magnification: ×5000 and ×10,000) (scale bar: 500 nm); the red arrow indicates the rupture of the mitochondrial cristae, and the black arrow represents the swelling of the mitochondria. (**B**–**G**) Quantitative analysis of mitochondrial parameters: perimeter (**B**), mitochondrial area (**C**), aspect ratio (**D**), cristae density per μm^2^ (**E**), cristae junction distance (**F**), and mitochondrial number per μm^2^ (**G**) (*n* = 6). All data are presented as mean ± SD. Statistical analysis was performed with one-way ANOVA. ** *p* < 0.01, *** *p* < 0.001.

First, to evaluate mitochondrial structural changes, we performed transmission electron microscopy (TEM). As shown in the TEM images (Figure 5A), cardiomyocytes in the DOX group exhibited obvious mitochondrial structural abnormalities, including mitochondrial swelling and reduced or absent cristae. In contrast, treatment with CDPs reversed DOX-induced mitochondrial matrix swelling and alleviated cristae shortening and reduction (Figure 5B–F). Additionally, DOX treatment led to a significant decrease in mitochondrial abundance, whereas CDPs administration restored mitochondrial numbers to near-normal levels (Figure 5G).

**Figure 6:**
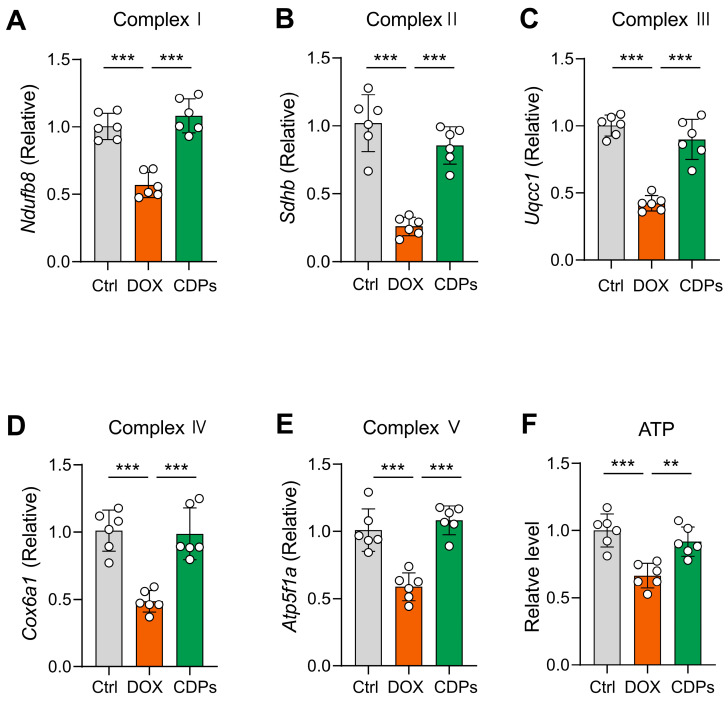
CDPs attenuate DOX-induced impairment of mitochondrial oxidative phosphorylation (OXPHOS) function. (**A**–**E**) Relative mRNA expression levels of mitochondrial respiratory chain complex-related genes (*n* = 6); (**F**) ATP levels in heart tissue (*n* = 6). All data are presented as mean ± SD. Statistical analysis was performed with one-way ANOVA. ** *p* < 0.01, *** *p* < 0.001.

Given that damaged mitochondrial structure can inhibit mitochondrial respiratory chain function [35,36,37,38], we further assessed the mRNA expression of mitochondrial respiratory chain complex markers via reverse transcription–quantitative polymerase chain reaction (RT-qPCR). The results showed that DOX induction impaired mitochondrial respiratory function, whereas CDPs treatment mitigated this imbalance and restored respiratory chain activity (Figure 6A–E).

Mitochondria are the major sites of ATP production in cells; thus, measuring ATP content can directly reflect mitochondrial energy metabolism status [39,40,41]. As anticipated, DOX treatment significantly decreased cardiac ATP concentrations, which indicates impaired oxidative phosphorylation (OXPHOS) (Figure 6F). Collectively, these results suggest that CDPs treatment alleviates DOX-induced cardiotoxicity, and this protective effect is associated with CDPs’ ability to improve mitochondrial structural abnormalities and restore compromised mitochondrial respiratory function.

## 4. Discussion

Currently, dexrazoxane is clinically widely used for the alleviation of DIC; nonetheless, it exhibits notable adverse effects, such as gastrointestinal reactions and bone marrow suppression [42,43]. Therefore, exploring safe and effective adjuvant therapeutic agents for DIC holds significant clinical importance. In our experiment, CDPs work through a dual synergistic mechanism: directly eliminating reactive oxygen species and restoring the structure and respiratory function of mitochondria to prevent the cardiac toxicity induced by doxorubicin. Additionally, they have good potential for clinical translation. The effective dose (200 mg/kg in mice ≈ 974 mg/day for 60 kg human, HED using Km = 12.3) is achievable with current Cistanche extracts already sold as health supplements [44]. Their natural source and unobserved toxicity highlight their potential as a safe adjunctive drug. Future trials should verify the preclinical findings and optimize the dosage.

The core pathological process of DOX-induced cardiotoxicity is pathological cardiac remodeling, characterized by reactivation of fetal genes during embryogenesis and myocardial fibrosis [45,46]. Natriuretic peptides (*Anp*) and brain natriuretic peptide (*Bnp*) are core molecular markers reflecting the severity of ventricular remodeling [47,48,49]. This study found that CDPs intervention significantly downregulated the mRNA levels of *Anp* and *Bnp* in the myocardium of DOX-induced heart failure mice.

The Sirius Red staining showed that the myocardial fibrosis area in the CDPs-treated group was significantly reduced compared to the model group [50]. As a key manifestation of pathological cardiac remodeling, the essence of myocardial fibrosis is the imbalance between collagen synthesis and degradation in the myocardial interstitium [51]. Type I collagen α1 chain (*Col1a1*) and type V collagen α1 chain (*Col5a1*) are the main components of the myocardial collagen network [50,51]. Under DOX induction, the expression of *Col1a1* and *Col5a1* significantly increased, which is a compensatory repair of the body to myocardial injury, aiming to maintain the integrity of the myocardial structure by enhancing collagen deposition [52]; however, the persistent cytotoxicity of DOX will disrupt the compensatory balance, leading to excessive collagen deposition, increasing the stiffness of the ventricular wall and impairing diastolic function [53,54], thereby exacerbating systolic dysfunction and forming a vicious cycle of heart failure progression.

Complementary to structural and molecular changes, CDPs also reduced serum levels of CK, CK-MB, and LDH—sensitive biomarkers of cardiomyocyte membrane integrity [55,56]. The reduction in these markers confirms that CDPs protect cardiomyocytes from DOX-mediated membrane damage, further linking their structural and molecular effects to preserved cardiac function.

Oxidative stress is not merely an upstream trigger but a core driver of DOX-induced cardiotoxicity, permeating the entire pathological process of myocardial damage [7]. Myocardial cells, characterized by high oxygen consumption and abundant mitochondria, are inherently vulnerable to oxidative damage—a vulnerability exacerbated by DOX, which selectively accumulates in myocardial mitochondria. Within mitochondria, DOX undergoes redox cycling to generate explosive ROS [57], which rapidly overwhelms the endogenous antioxidant defense system of myocardial cells. In addition, ROS attack polyunsaturated fatty acids in the myocardial cell membrane, generating malondialdehyde [58,59]. These lipid peroxides further crosslink with myocardial structural proteins, thereby impairing myocardial contractile function.

CDPs have been reported to protect against various oxidative stress-related pathologies [20,31]. The structural characteristics of CDPs provide critical insights into their potential antioxidant activity [25,31]. CDPs fall into the category of low-/medium–low-molecular-weight polysaccharides and have higher antioxidant activities than higher-molecular-weight polysaccharides, because LMWPs do have their reductive groups (hydroxyl and amino groups) accessible to reactive radicals and oxidants [60]. FTIR spectra further confirmed functional groups critical for antioxidant activity: the broad band at 3425 cm^−1^ (O-H stretching) indicates abundant hydroxyl groups, the peak at 1631 cm^−1^ (C=O stretching) facilitates electron transfer with free radicals, and the band at 1025 cm^−1^ (C-O-C vibration) verifies pyranose rings that stabilize the polysaccharide structure [22]. These structural features collectively support CDPSs’ potential as effective antioxidants.

Given that mitigating oxidative stress-induced myocardial damage is a core strategy for alleviating DOX-induced cardiotoxicity and subsequent heart failure, the present study aimed to evaluate whether CDPs could preserve cardiac function by interrupting the oxidative stress-mediated myocardial injury cascade. Our results showed that CDPS intervention robustly reversed DOX-induced oxidative perturbations: it restored the activities of SOD and GSH-Px in both myocardial tissue and serum, while simultaneously reducing MDA levels in these two matrices. Importantly, CDPs’ antioxidant effects extend beyond the myocardium to the systemic circulation—a finding that holds notable clinical significance. This is because DOX-induced oxidative stress is not organ-specific but systemic in nature, and such systemic oxidative damage contributes to the development of multi-organ toxicity [2]; thus, CDPs’ ability to modulate systemic redox status may not only protect the heart but also mitigate DOX’s off-target toxic effects—offering a potential strategy to improve the safety of DOX-based chemotherapy.

Oxidative stress is not only an upstream trigger of DOX cardiotoxicity but also a key mediator of mitochondrial damage [6,7]. Thus, we further explored CDPs effects on mitochondrial structure and function. To validate these pathological changes, we performed TEM analysis on cardiac tissue from DOX-treated mice. Consistent with the aforementioned mechanistic deductions, TEM images revealed severe mitochondrial damage in the DOX group. In contrast, pre-treatment with CDPs markedly ameliorated these structural impairments: relative to the DOX-treated group, CDPs intervention effectively restored the mitochondrial number, normalized mitochondrial morphological parameters (area and aspect ratio), increased cristae density, and reduced cristae spacing [61,62]. These structural improvements are of pivotal importance for preserving mitochondrial function: by restoring cristae density and spacing, CDPs may facilitate the maintenance of respiratory complex integrity and optimize electron transfer efficiency; concurrently, the restoration of mitochondrial number and morphology helps ensure sufficient oxidative phosphorylation capacity to meet the high energy demands of cardiomyocytes [6,37,63]. We found that CDPs significantly improved the damaged mitochondrial structure that was disrupted by DOX, which likely explains their ability to restore the expression of respiratory chain complex genes. These complexes are critical for electron transport and OXPHOS; their downregulation by DOX is a major cause of ATP depletion. Consistent with this, CDPs restored cardiac ATP levels, reversing DOX-induced OXPHOS impairment.

Nonetheless, this study has some limitations that should be acknowledged. First, previous studies have reported that male and female mice exhibit significant differences in susceptibility to dox-induced cardiotoxicity, with male mice showing higher vulnerability [64,65]. Consequently, we exclusively used male mouse models throughout the entire study. While this approach helped control for potential confounding factors related to female-specific hormonal fluctuations, it may introduce limitations to the study’s internal validity and inherently restricts the generalizability of the findings across both male and female populations. Second, the current experiment only adopted a single-dose intervention regimen. Future studies should optimize the dose design by establishing multiple dose gradients; this will help clarify the dose–effect relationship and further enhance the scientific rigor and translational potential of the research outcomes.

## 5. Conclusions

**Figure 7:**
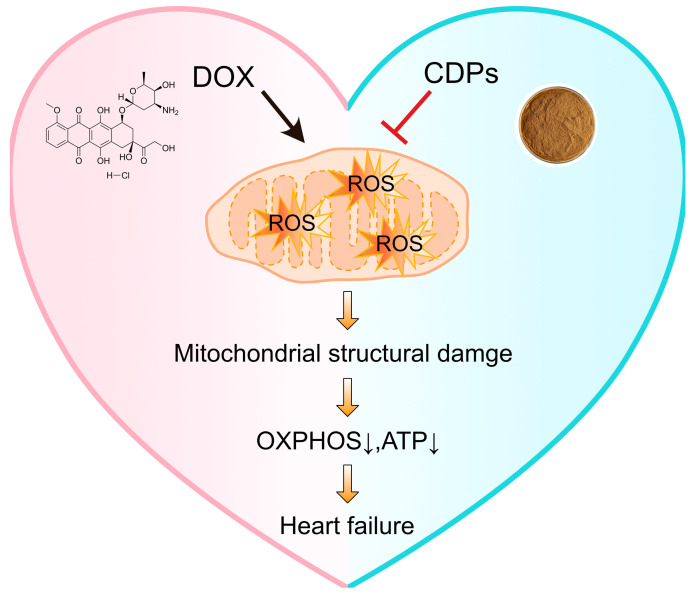
Schematic illustration of CDPs against DOX-induced cardiotoxicity. DOX triggers the overproduction of ROS in mitochondria, leading to mitochondrial structural damage. This damage subsequently impairs OXPHOS and reduces ATP production, ultimately contributing to heart failure. In contrast, CDPs inhibit DOX-induced ROS generation, thereby alleviating mitochondrial damage, maintaining OXPHOS function and ATP levels, and preventing heart failure.

This study confirms that CDPs can significantly alleviate DOX-induced cardiotoxicity in mice and protect cardiac function. The core of their mechanism of action is manifested in the following aspects: Firstly, CDPs directly suppress the imbalance of myocardial oxidative stress by enhancing the activity of antioxidant enzymes (SOD, GSH-Px) and reducing the levels of oxidative damage products (MDA). Secondly, they reduce the source of reactive oxygen species (ROS) generation by improving mitochondrial morphology and energy metabolism function—thereby indirectly blocking the amplification effect of oxidative stress (Figure 7). These results suggest that CDPs exhibit considerable prospects for application in the development of functional foods or clinical adjuvants targeting DOX-induced cardiotoxicity, and further clarify the key value of targeted oxidative stress regulation in the intervention of DOX-induced cardiotoxicity.

## Data Availability

Data are contained within the article and Supplementary Materials.

## Supplementary Materials

The following supporting information can be downloaded at https://www.mdpi.com/article/10.3390/antiox14121461/s1, Figure S1:Melting Curve Analysis; Figure S2: Efficiency analysis; Figure S3: Monosaccharide composition, HPGPC and FTIR analysis of CDPs; Table S1: Primers used for the PCR assays in this study; Table S2: Monosaccharide compositions (%) of CDPs; Table S3: Molecular Weight Parameters of CDPs Fractions.

## Abbreviations

ATP: adenosine triphosphate; *Anp*: atrial natriuretic peptide; *Bnp*: brain natriuretic peptide; BW: body weight; CD: *Cistanche deserticola* Y.C. Ma; CDPs: *Cistanche deserticola* polysaccharide; CK: creatine kinase; CK-MB: creatine kinase-MB; CSA: cross-sectional area; CO: cardiac output; *Col1a1*: type I collagen α1 chain; *Col5a1*: type V collagen α1 chain; DOX: Doxorubicin; ETC: electron transport chain; FTIR: Fourier transform infrared spectroscopy; Glc: glucose; Gal: galactose; GSH-Px: glutathione peroxidase; HF: heart failure; HW: heart weight; HPLC: high-performance liquid chromatography; HPGPC: high-performance gel permeation chromatography liquid chromatography; HE: hematoxylin–eosin; LDH: lactic dehydrogenase; LVEF: left ventricular ejection fraction; LVFS: left ventricular fraction shortening; LVID; s: left ventricular internal dimension at systole; LVESV: left ventricular end-systolic volume; Mw: molecular weight; Mn: the number mean molecular weight; Man: mannose; MDA: malondialdehyde; OXPHOS: oxidative phosphorylation; PMP: 1-phenyl-3-methyl-5-pyrazolone; ROS: reactive oxygen species; SOD: superoxide dismutase; TEM: transmission electron microscopy; TL: tibia length; WGA: wheat germ agglutinin.
