# Supplementary material for: Cistanche deserticola Polysaccharides Protect Against Doxorubicin-Induced Cardiotoxicity via Antioxidant and Mitochondrial Mechanisms
Source: Antioxidants (Basel). 2025 Dec 5;14(12):1461. doi: 10.3390/antiox14121461 (PMC12729767; doi:10.3390/antiox14121461)
Supplement: Supplementary file 1 [file antioxidants-14-01461-s001.zip › Supplemental figures.pdf]

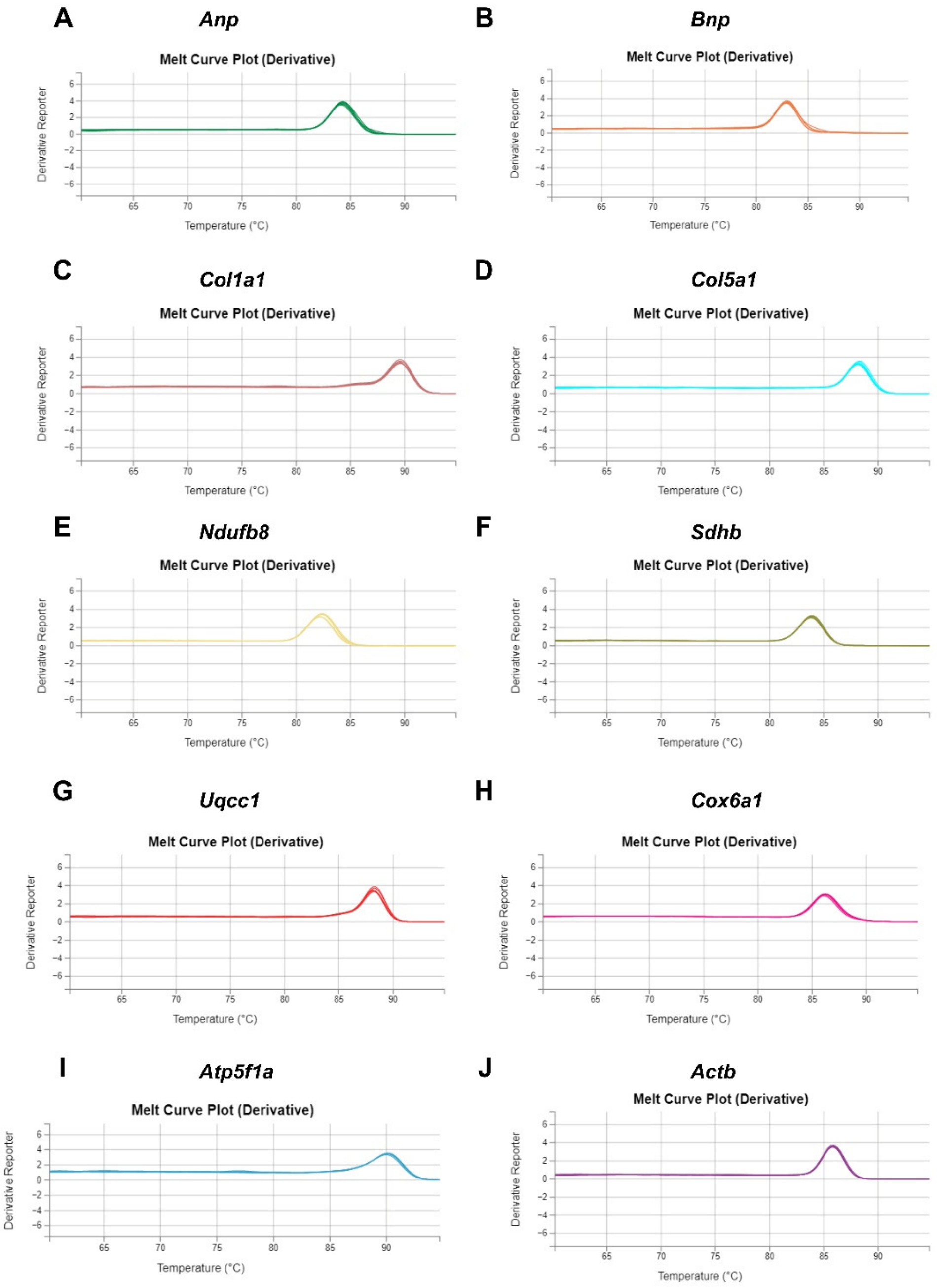

Figure S1. Melting Curve Analysis. All primer melting curve peaks are sharp and uniform, with no small peaks in the low-temperature region. This indicates that the primers have excellent specificity, no primer dimers are formed, and there is no non-specific amplification.

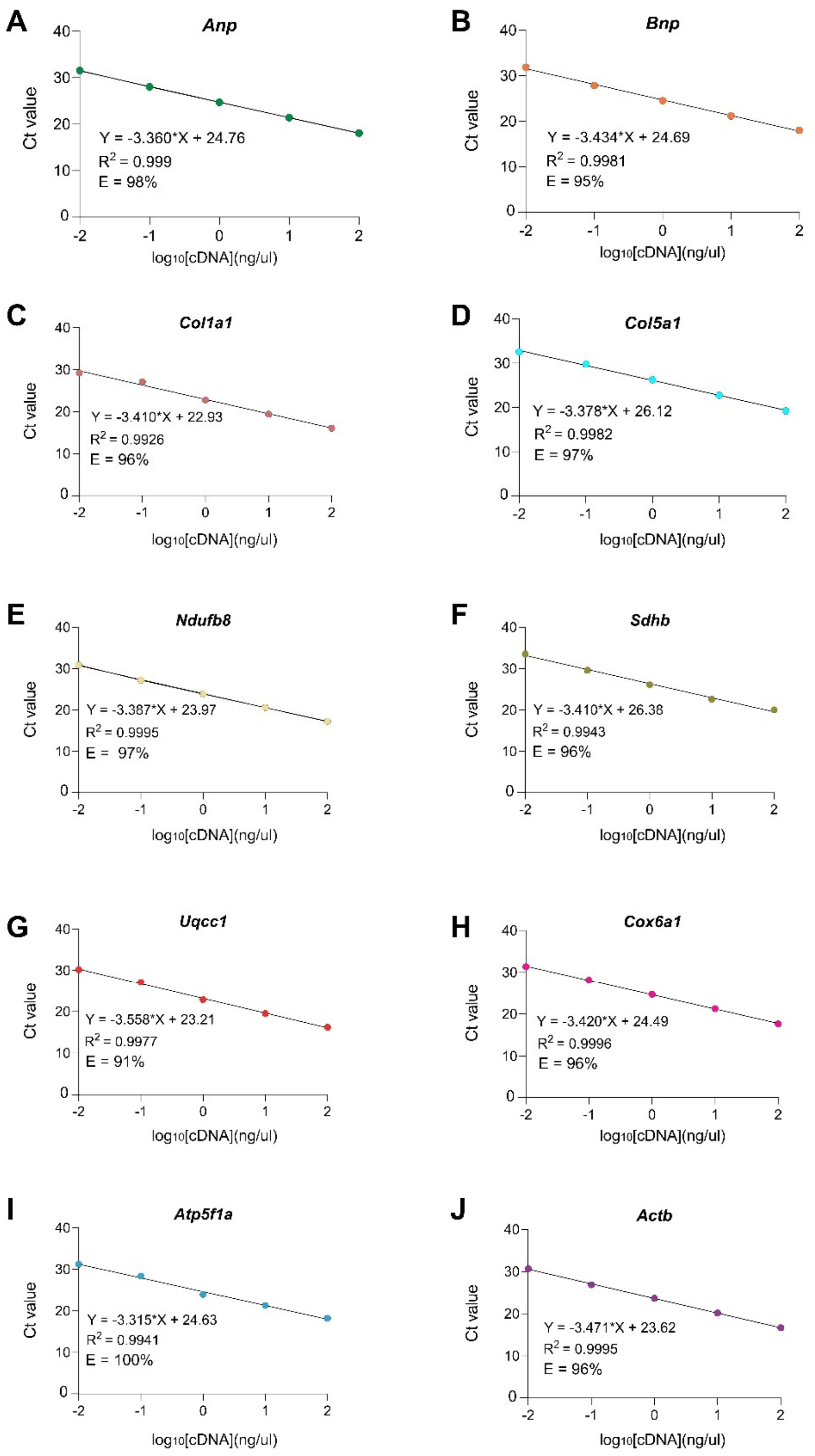

Figure S2. Efficiency analysis. The efficiency of all genes ranged from 90% to 110%, indicating good RT-qPCR amplification efficiency.

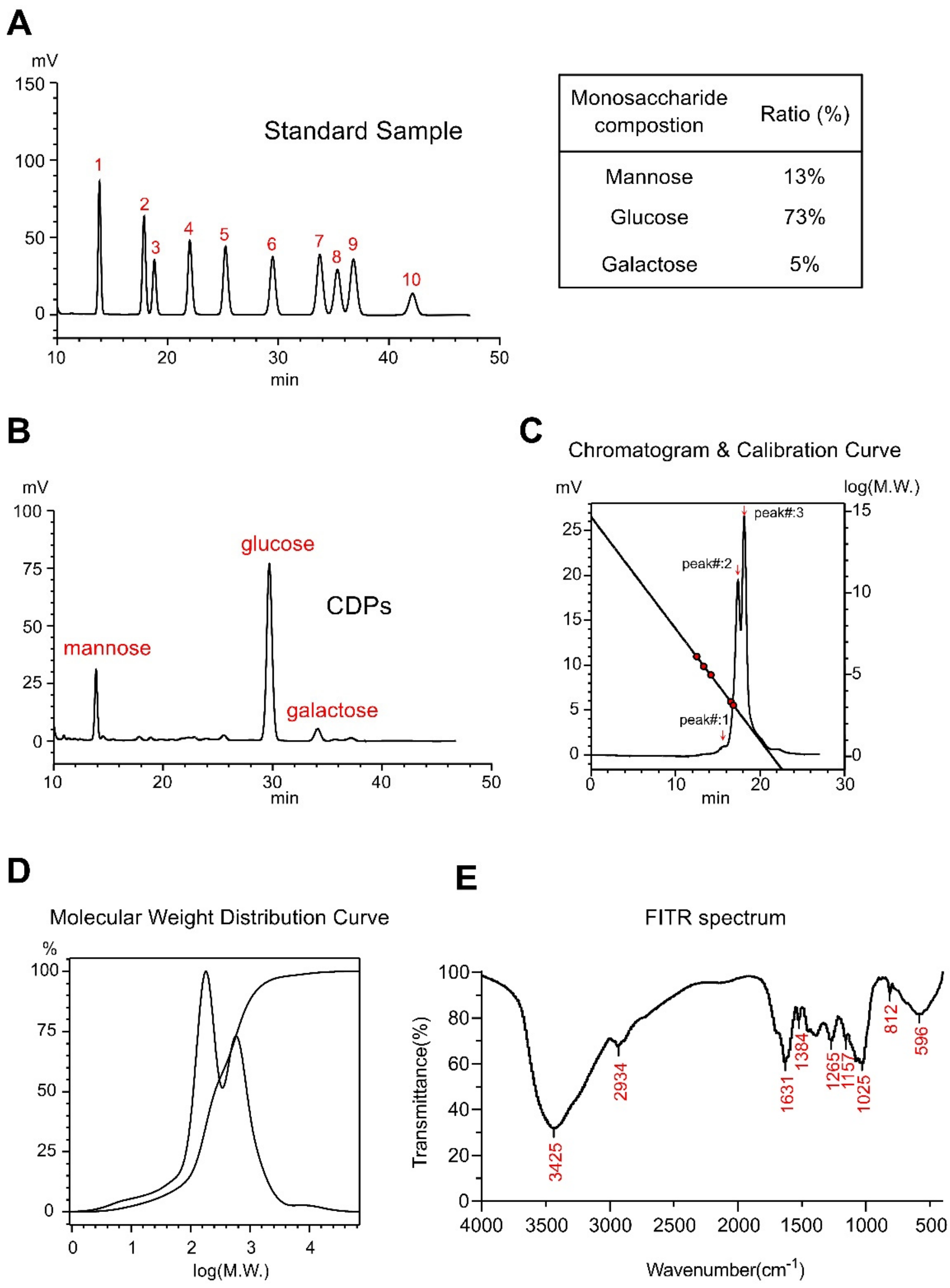

Figure S3. Monosaccharide composition, HPGPC and FTIR analysis of CDPs. (A) Standard monosaccharides; (B) the composition of CDPs by HPLC analysis: 1: mannose, 2: ribose, 3: rhamnose, 4: glucuronic acid, 5: galacturonic acid, 6: glucose, 7: galactose, 8: xylose, 9: arabinose, 10: fucose; (C) chomatogram & calibration curve of CDPs; (D) molecular weight distribution curve of CDPs; (E) FTIR spectrum of CDPs.
