# Supplementary material for: Cistanche deserticola Polysaccharides Protect Against Doxorubicin-Induced Cardiotoxicity via Antioxidant and Mitochondrial Mechanisms
Source: Antioxidants (Basel). 2025 Dec 5;14(12):1461. doi: 10.3390/antiox14121461 (PMC12729767; doi:10.3390/antiox14121461)
Supplement: Supplementary file 1 [file antioxidants-14-01461-s001.zip › Supplemental Tables.pdf]

Table S1. Primers used for the PCR assays in this study.

Gene	GeneBank sequence number	Forward primer Sequence (5'->3')	Reverse Primer Sequence (5'->3')	Product length (bp)
Anp	NM_008725	GCTTCGGGGGTAGGATTGAC	GAGGCAAGACCCCACTAGAC	268
Bnp	NM_008726	GGAAGTCCTAGCCAGTCTCCAGAG	GCCTTGGTCCTTCAAGAGCTGTC	108
Col1a1	NM_007742	CCTCAGGGTATTGCTGGACAAC	CAGAAGGACCTTGTTTGCCAGG	115
Col5a1	NM_015734	CTTCGCCGCTACTCCTGTTC	CCCTGAGGGCAAATTGTGAAAA	110
Ndufb8	NM_026061	CGCCAAGAAGTATAACATGCGAG	CCTCTCATGCTGTGATCGGTTG	106
Sdhb	NM_023374	TGCGGACCTATGGTGTTGGATG	CCAGAGTATTGCCTCCGTTGATG	133
Uqcc1	NM_018888	CAGCCTGTTGAAGAGAAGGTCG	CGAAGTCAGTCTTCTCCACGCA	142
Cox6a1	NM_007748	TCAACGTGTTCCTCAAGTCGC	AGGGTATGGTTACCGTCTCCC	115
Atp5f1a	NM_007505	TGGTGAAGAGACTGACGGATGC	TCAAAGCGTGCTTGCCGTTGTC	150
Actb	NM_007393	GGTACCACCATGTACCCAGG	AAACGCAGCTCAGTAACAGTC	249

Table S2. Monosaccharide compositions (%) of CDPs

Monosaccharide compositions	Molar ratio	Ratio (%)
mannose	0.50	13%
Ribose	0.06	2%
rhamnose	0.06	2%
glucuronic acid	0.04	1%
galacturonic acid	0.07	2%
glucose	2.81	73%
galactose	0.19	5%
xylose	0.04	1%
arabinose	0.08	2%

Table S3. Molecular Weight Parameters of CDPs Fractions

Fraction	Retention Time (min)	Mn (Da)	Mw (Da)	PDI (Mw/Mn)
Peak#1	14.350–15.950	10316	13314	1.29
Peak#2	15.950–17.717	665	902	1.36
Peak#3	17.717–21.483	53	156	2.95
